# Evaluation of the Potential Impact of In Silico Humanization on V_H_H Dynamics

**DOI:** 10.3390/ijms241914586

**Published:** 2023-09-26

**Authors:** Carla Martins, Julien Diharce, Aravindan Arun Nadaradjane, Alexandre G. de Brevern

**Affiliations:** 1 Université Paris Cité and Université de la Réunion and Université des Antilles, INSERM, BIGR, DSIMB, F-75014 Paris, France; cmartins@insa-toulouse.fr (C.M.); julien.diharce@u-paris.fr (J.D.); 2 Université Paris Cité and Université de la Réunion and Université des Antilles, INSERM, BIGR, DSIMB, F-97715 Saint Denis Messag, France

**Keywords:** secondary structure, sequence structure relationship, structural alphabet, antibodies, nanobodies, molecular dynamics simulations, frameworks

## Abstract

Camelids have the peculiarity of having classical antibodies composed of heavy and light chains as well as single-chain antibodies. They have lost their light chains and one heavy-chain domain. This evolutionary feature means that their terminal heavy-chain domain, VH, called V_H_H here, has no partner and forms an independent domain. The V_H_H is small and easy to express alone; it retains thermodynamic and interaction properties. Consequently, V_H_Hs have garnered significant interest from both biotechnological and pharmaceutical perspectives. However, due to their origin in *camelids*, they cannot be used directly on humans. A humanization step is needed before a possible use. However, changes, even in the constant parts of the antibodies, can lead to a loss of quality. A dedicated tool, Llamanade, has recently been made available to the scientific community. In a previous paper, we already showed the different types of V_H_H dynamics. Here, we have selected a representative V_H_H and tested two humanization hypotheses to accurately assess the potential impact of these changes. This example shows that despite the non-negligible change (1/10th of residues) brought about by humanization, the effect is not drastic, and the humanized V_H_H retains conformational properties quite similar to those of the camelid V_H_H.

## 1. Introduction

**Figure 1:**
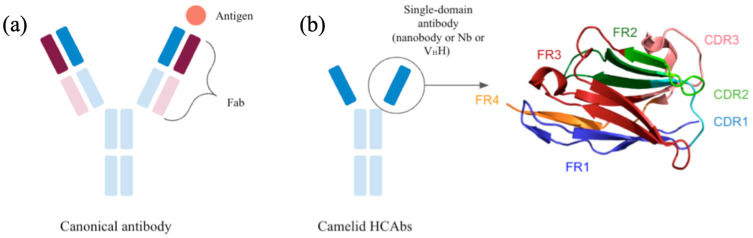
*Antibodies*. Schematic representation of (**a**) a canonical immunoglobulin (the Fab domains contain the VH and VL regions that bind to the paratope), and (**b**) A heavy chain only antibody (HCAB) with a zoom on V_H_H domain. The different domains are in similar color for a simple comparison. The four frameworks (FRs) are FR1 (purple), FR2 (green), FR3 (red), and FR4 (orange), while the complementary determining regions (CDRs) are CDR1 (cyan), CDR2 (light green), and CDR3 (pink).

For the past 40 years, antibodies have played a major role in biotherapy and in detection and imaging biotechnology [1,2]. A typical antibody is composed of two similar dimers, each consisting of two chains, a heavy and a light chain (see Figure 1a). The antigen-binding interface is distributed between the N-terminal variable domains of the heavy (VH) and light (VL) chains. However, analysis of antibodies in camelid species (consisting of vicuña, alpaca, llama, camel, and dromedary) has identified a second class of functional antibodies called heavy-chain antibodies (HCAbs). These specific antibodies consist only of heavy chains and lack the first domain of the constant region (CH1) [3].

Unlike classical antibodies, HCAbs bind antigens only via the variable domain of the heavy chain (VH), also called V_H_H or Nanobody^®^, a name registered by AbLynx, a Sanofi company dedicated to these proteins. V_H_H has a structure similar to that of VH and VL in classical antibodies. It is composed of a series of frameworks (FRs), which are the most highly conserved regions [4], interspersed with hypervariable regions called complement determining regions (CDRs, see Figure 1b). But some differences from classical immunoglobulins have been underlined. The FR2 and CDR3 regions are significantly larger on average. Compared to conventional entire immunoglobulins, V_H_Hs are 20 times smaller, with a molecular weight of 90 kDa, making them easier to produce. Moreover, their affinity is close to that of conventional antibodies, allowing them to reach other epitopes [5,6].

Their size and their proximity, in terms of identity rate [7,8], to conventional VH of human antibodies make them suitable for use in humans due to their low immunogenicity [9]. V_H_Hs are also more stable, highly soluble, and can refold after denaturation when compared to conventional antibodies [10,11,12]. All of these properties make V_H_Hs particularly attractive as potential therapeutic tools [13,14,15,16,17], e.g., against the severe acute respiratory syndrome coronavirus 2 (SARS-CoV-2) receptor-binding domain [18,19,20]. In 2018, V_H_Hs were used for the first time in the therapeutic setting with the introduction of a treatment called caplacizumab for patients with acquired thrombotic thrombocytopenic purpura. This usually fatal disease is characterized by microangiopathic hemolytic anemia and severe thrombocytopenia [21]. Recently, V_H_H Ozoralizumab (Nanozora), a novel tumor necrosis factor (TNF) inhibitor, was approved in Japan for the treatment of rheumatoid arthritis. It inhibits TNF action through two human TNFα-binding domains [22,23,24]. However, V_H_Hs produced by *Camelidae* family members need to be adapted for humans by increasing the similarity of antibodies of non-human origin to human antibodies in order to prevent an unwanted immune response [25,26]. Consequently, it is crucial to understand the impact of humanization on the V_H_H structure and the dynamics that govern its interaction with its paratope. To this end, a recent in silico tool allowing the humanization step to be carried out has been made available to the scientific community; it is called Llamanade (https://github.com/sangzhe/Llamanade/blob/main/README.md, accessed on 29 May 2023) [27]. Llamanade can use not only the V_H_H sequence but also an V_H_H experimental structure. Unfortunately, in practice, the structure of the V_H_H is often not available. It is therefore necessary to model it, a complex step [28,29]. Deep learning approaches are the best known today to predict the structure of these particular proteins, as brought forth by Llamanade, which allows the incorporation of the NanoNet tool [30], a very efficient approach that we have already extensively tested [31]. The binding to its partner then depends strongly on its dynamics, which are also difficult to understand [32]. Llamanade has been shown to be useful against SARS-CoV-2 using the sequence and/or structure of the V_H_H in question.

Here, we decided to investigate the potential effect of humanization on the dynamics of V_H_H. We therefore took an experimentally resolved V_H_H structure and used Llamanade with two scenarios: (i) providing only the sequence and using NanoNet to build a structural model for the humanized V_H_H, and (ii) providing the true structure to Llamanade for the humanized V_H_H. Molecular dynamics simulations would provide insight into the structural consequences of the humanization step.

## 2. Results and Discussion

### 2.1. V_H_H Structure

**Figure 2:**
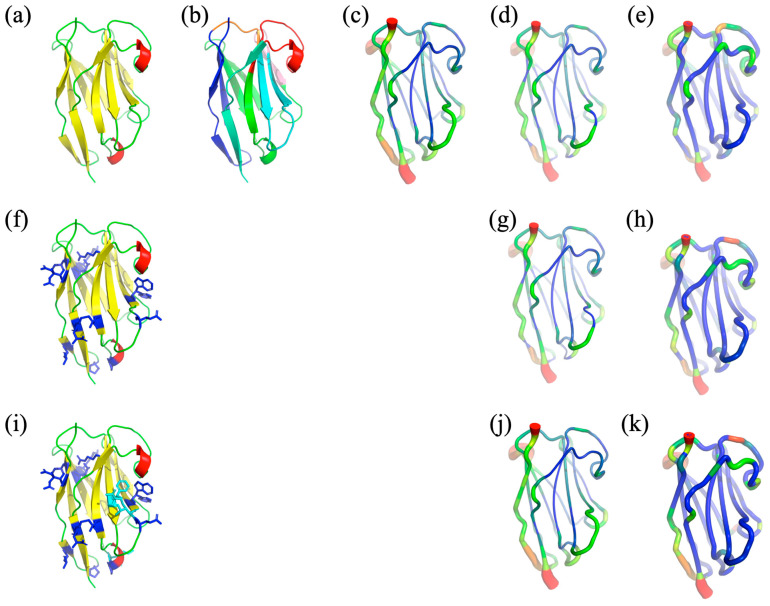
*HL6 V_H_H and the structural models*. (**a**–**e**) HL6 V_H_H [34], (**f**–**h**) V_H_H structural model from the X-ray structure (V_H_H-h_str_), (**i**–**k**) V_H_H structural model from the sole information of the sequence, modeled by NanoNet (V_H_H-h_seq_). (**a**) Secondary structure assignment, (**b**) FRs and CDRs visualization (FR1: positions 1 to 25 in blue, CDR1: 26 to 30 in orange, FR2: 31 to 48 in cyan, CDR2: 49 to 56 in pink, FR3: 57 to 93 in green, CDR3: 94 to 109 in red, and FR4, 110 to 121 in light green), (**f**,**i**) variant positions with stick representations (in blue and cyan, respectively), (**c**) B-factors, (**d**,**g**,**j**) RMSF, (**e**,**h**,**k**) *N*_eq_ value visualization in the structure and structural models. For (**c**–**e**,**g**,**h**,**j**,**k**), colors ranging from dark blue for the minimal value to green, orange, and finally red for the maximal value.

The selected V_H_H structure noted in HL6 was taken from PDB [33] id 1OP9 [34] (see Figure 2a), and it is in complex with human lysozyme. It had a good resolution of 1.86Å with no missing residues and excellent quality of all other parameters such as R-free, Ramachandran outliers, or side-chain quality. It is 121 residues long and is composed of a series of β-strands forming the classic β-sheet of V_H_Hs and VHs/VLs. It therefore has FRs of classical sizes (FR1 position 1–25, FR2 position 31–48, FR3 position 57–93, and FR4 position 110–121) and classic CDRs of V_H_Hs (CDR1 position 26–30, CDR2 position 49–56, and CDR3 position 94–109). V_H_H HL6 is a perfect candidate for our analysis.

### 2.2. Humanisation

**Figure 3:**
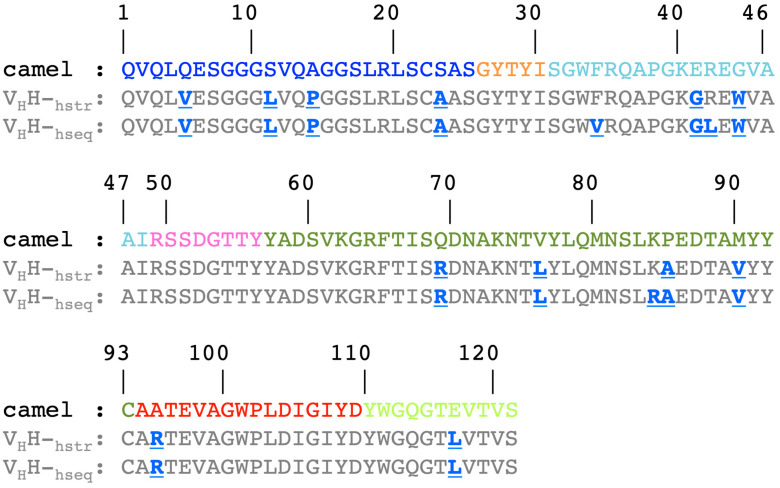
*V_H_H sequences*. Provided is the sequence for the camel V_H_H (noted camel), the V_H_H structural model from the X-ray structure (noted V_H_H-h_str_), and the V_H_H structural model from the sole information of the sequence, modeled by NanoNet (V_H_H-h_seq_). The colors are the same as for Figure 2b; the variants in V_H_H-h_str_ and V_H_H-h_seq_ are noted in bold underlined blue.

The two humanization scenarios are realized by using the sequence or the structure. This study allows for the obtaining of two structural models corresponding to the humanized V_H_H (see Figure 3). The process of humanization consists of a series of point mutations without insertion or deletion; the humanized sequences therefore have the same length as the camelid sequences. The first humanized V_H_H is generated from the sole information of the sequence; 15 mutations are identified when compared to the HL6 sequence; it is named V_H_H-h_seq_. The second one is generated from its structure (PDB id 1OP9 [33]) and presents 12 mutations; it is named V_H_H-h_str_. These 12 mutations are also found in V_H_H-h_seq_. The majority of mutations are identified within the FRs: four on FR1 (Q5V, S11L, A14P, S23A), two on FR2 (E41G and G44W), and four on FR3 (Q69R, V76L, P85A, M90V). One mutation is present on CDR3 (A95R) and one on FR4 (E116L). The three additional mutations present only on V_H_H-h_seq_ are found at FR2 (F34V, R42L) and FR3 (K84R). Hence, 9.9% and 12.4% of the residues have been modified during the humanization process (see Figure S1).

The principle of the two scenarios is very similar, with Llamanade using a model generated from the sequence to optimize the humanization of V_H_H-h_seq_, whereas for V_H_H-h_str_ it already has access to the structure. The three residues treated differently are due to the fact that the models are not identical to the experimental structures, hence the slightly different proposals.

As expected, the two new sequences are closer to human immunoglobulin than the original camelid one. A simple search for human antibody sequences with PSI-BLAST [35] can be performed with humanized V_H_Hs. Hence, we found one of the human SARS-COV-2 antibodies (Genbank id QWS71009.1 [36]) sharing a significant sequence identity of 78% and a sequence similarity of 87% with the modified sequences. All point mutations can be easily compared; only two mutated positions are not found in this human SARS-COV-2 antibody, position 5 in FR1 (L5) and position 95 in CDR3 (K95).

By using our previous results and other analyses on the distribution of amino acids in V_H_Hs [7,8,32], it is possible to quantify these changes as (i) predominant (i.e., majority or even exclusive in V_H_Hs), (ii) same frequency (i.e., having equivalent observed frequencies in V_H_Hs), (iii) minority (i.e., they have a very low observed frequency, clearly not in the majority for this position in the V_H_Hs), and (iv) never seen in any of the V_H_Hs. Four positions go to an amino acid that is predominant in V_H_Hs (S11L, S23A, Q69R, M90V), two mutations were made to a type of residue that has the same frequency in V_H_Hs (Q5V, Q14P), four mutations are found in a minority of V_H_Hs (E41G, G44W, V76L, E116L), and four mutations are never seen in V_H_Hs (P85A, F34V, R42L, K84R). So, for this last case, it encompasses all three specific positions of V_H_H-h_seq_.

Furthermore, 20 years ago, Muyldermans [37] described five positions specific to V_H_H in relation to VH. They are all modified here in V_H_H-h_seq_, namely S11L (also S11L with Kabat numbering [38,39,40]), F34V (F37V), E41G (E44G), R42L (R45L), and G44W (G47W). Nevertheless, the use of the 3D structure for V_H_H-h_str_ means that two of these mutations (namely F34V and R42L) are not used, underlying the specificity of having a real structure to guide the humanization, as noted in [27].

The authors of Llamanade have noted a series of experimental positions (known as hotspots) that are preferentially humanized [27]. These include FR1 (A14P), FR2 (E41G and G44W), FR3 (V76L and P85A), and FR4 (E116L). Thus, more than half of the mutations are specific to this particular V_H_H, with three on FR1 (Q5V, S11L, and S23A), two on FR3 (Q69R and M90V), and one on the border with CDR3 (A95R). Similarly, the three additional mutations present only on V_H_H-h_seq_ are not hotspots either.

The V_H_H humanized structural models are close to the experimental structure (the root mean square deviation on Cα is of 1.08 Å for V_H_H-h_seq_ but only 0.71 Å for V_H_H-h_str_, and corresponding TM-scores [41] are of 0.90 and 0.92, resp. (see structure in Figure 2a and models in Figure 2h,i). Thus, (i) the structural approximation seems of better quality, and (ii) the number of identified mutations is lower when using the structure. We can hypothesize (and it seems logical) that the results generated by Llamanade could be more reliable with the use of the structure than just with the sequence.

### 2.3. Analysis of HL6 Molecular Dynamics Simulations

**Figure 4:**
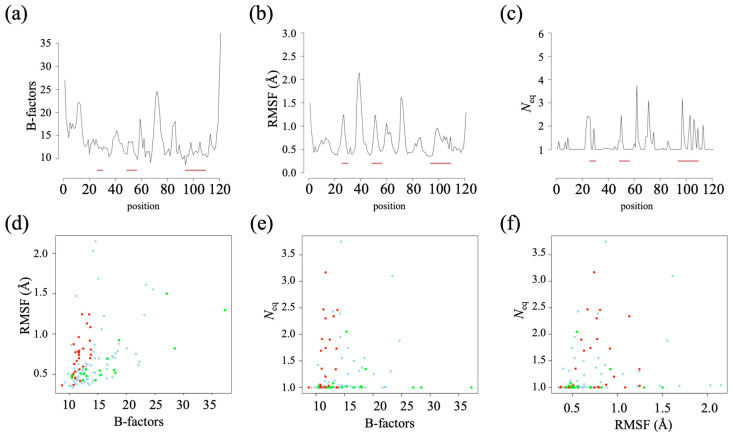
*Analysis of the structure and dynamics of HL6 V_H_H*. (**a**) B-factors, (**b**) root mean square fluctuation (RMSF), (**c**) number of equivalent (*N*_eq_) of HL6 V_H_H, (**d**) comparison between B-factors and RMSF, (**e**) comparison between B-factors and *N*_eq,_ and (**f**) comparison between RMSF and *N*_eq_. (**a**–**c**) The red lines correspond to CDRs’ positions. (**d**–**f**) in red color positions associated with CDRs, in green the extremities, and in blue the rest of FRs.

As often observed, high B-factor values (obtained from the crystallographic data) indicate that the N- and C-terminal extremities have the highest flexibility (see Figure 2c and Figure 4a). As HL6 V_H_H is obtained in complex with lysozyme, its CDRs have lower B-factor values, while the highest values are seen in FR3 and also in FR1.

A root mean square deviation (RMSD) plateau is reached for all MD simulations at a steady state of 150 ns, indicating stable and reproducible independent dynamics.

In terms of root mean square fluctuation (RMSF, see Figure 2d and Figure 4b), the two most flexible regions are FR2 (RMSF higher than 2.0 Å) and the center of FR3 (RMSF of 1.6 Å). These regions correspond mainly to loops (connecting the β-strands of the FRs) located opposite the CDRs. CDR1 and CDR2 are then the most flexible regions (RMSF of 1.0 Å), followed by the beginning of FR3 and finally by CDR3. The β-strands are, as expected, associated with low RMSF values (often 0.5 Å). This V_H_H therefore has a dynamic corresponding to the second most common dynamic class of V_H_Hs (see [32]). The correlation between the B-factor and the RMSF is 0.44 (see Figure 4d), which is exactly what is classically observed [42].

The number of equivalent (*N*_eq_) measurements is quite different from the RMSF, which gives an overall assessment, whereas the *N*_eq_ is local (a protein block or PB has five residues). Analysis of the dynamics of the V_H_H shows that it is rather rigid (see Figure 2e and Figure 4c). Almost half (60/121) of the positions have a *N*_eq_ of 1.0 (no change in the PB during the dynamics), indicating total local rigidity, including the β-strands but also parts of the loops. The rest is not very flexible (51 positions still have a *N*_eq_ less than 2.0). Of the ten positions with a *N*_eq_ greater than 2.0, only three have a *N*_eq_ greater than 3.0 (a *N*_eq_ of 4.0 is the classical threshold for flexibility [43]). These positions correspond to FR3 (positions 62 and 72) and CDR3 (position 97). On average, CDRs are more deformable (CDR1: 1.45, CDR2: 1.27, and CDR3: 1.52) than FRs (FR1: 1.20, FR2: 1.04, FR3: 1.24, and FR4: 1.09). CDR3 is the most deformable one but also has a number of rigid regions with low *N*_eq_ (positions 94–96, 100–101, 104–105, 108). Analysis of the PB assignments (see Figure S2a,b) showed that the first most deformable region (position 62) is associated with a very unusual loop with the PB series *goxac*, where *x* are the PBs *p*, *k*, and *i* (this series is not associated with the classical loop [44]). The second one (position 97) is a very classical one with the PB series *kxop*, where *x* are PBs *l*, *n*, or *k* [44]. Similarly, the beginning of CDR3 is very classical; position 97 is very β-stranded PB.

As the B-factor and the RMSF are therefore methodologies taking into account the entire structure, it is logical to have a very low correlation of the *N*_eq_ with the former (0.22, see Figure 4e) and the latter (0.06, see Figure 4f), as previously seen [42].

### 2.4. Analysis of Humanized V_H_Hs Dynamics

**Figure 5:**
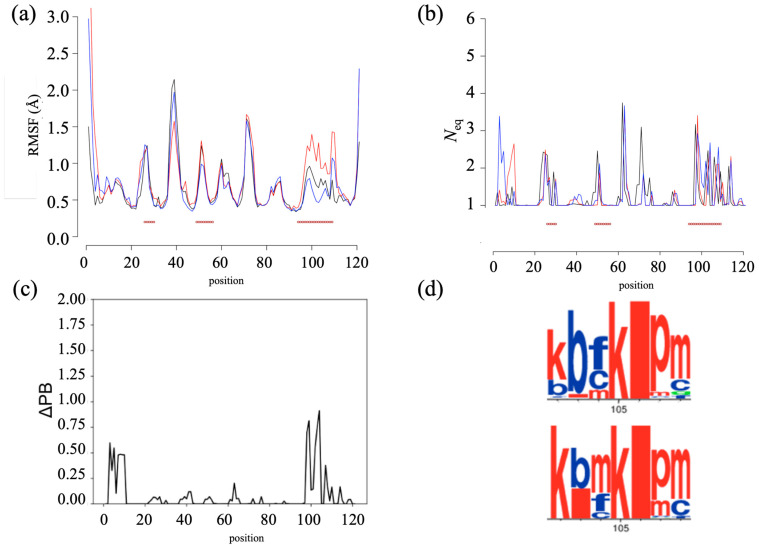
*Humanized V_H_Hs*. Flexibility analysis using (**a**) RMSF and (**b**) *N*_eq_ with V_H_H-h_seq_ in blue and V_H_H-h_str_ in red. The red lines correspond to CDRs’ positions. (**c**) ΔPB and zoom of (**d**) WebLogo between positions 102 and 108 (PBs in red are more associated to helical conformations and in blue to extended conformations).

The two humanized V_H_Hs differ by only three residues. The RMSF analysis shows a surprisingly similar trend for both humanized V_H_Hs (see Figure 5a). Excluding the extremities, only a small difference is observed in the FR2 regions (variation differences of 0.02 nm), and V_H_H-h_seq_ exhibits a significant peak in the CDR3 region (variation differences of 0.1 nm), indicating greater flexibility in CDR3.

The analysis with PBs blocks and *N*_eq_ (see Figure 5b) demonstrates more complex patterns. Overall, there is greater flexibility observed in the FR1, CDR1, FR3, and CDR3 regions. The V_H_H models are particularly rigid, with 52.9% and 47.9% with a *N*_eq_ of 1.0 and 92.6% and 90.1% with a *N*_eq_ of less than 2.0 for V_H_H-h_seq_ and V_H_H-h_str,_ respectively. Only two positions at any one time have a *N*_eq_ greater than 3.0. In both cases, positions 63 and 98 have a high *N*_eq_ (see Figure 5b, 3.62 and 3.67, then 3.41 and 2.93, respectively). Only the N-terminus shows clear variations.

To compare the conformations between the two humanized V_H_Hs, the ΔPB is calculated. The advantage of this measure is that it allows a direct comparison of the PB distributions observed during the dynamics. Identical *N*_eq_ values can correspond to different conformations. This analysis revealed a few conformational differences (see Figure 5c). For the FR1 and CDR3 regions, a conformational disparity of 25% (ΔPB of 0.50) is observed between the two humanized V_H_Hs. This disparity is illustrated by the WebLogo representation between positions 102 and 108 (see Figure 5d). V_H_H-h_seq_ shows the PB series *kbfkipm*, whereas V_H_H-h_str_ PB shows the PB series *kbmkipm*. This difference is worth highlighting, even though it is relatively small.

All these analyses show that the humanized V_H_Hs remain largely similar.

### 2.5. Comparing Wild-Type with Humanized V_H_Hs

**Figure 6:**
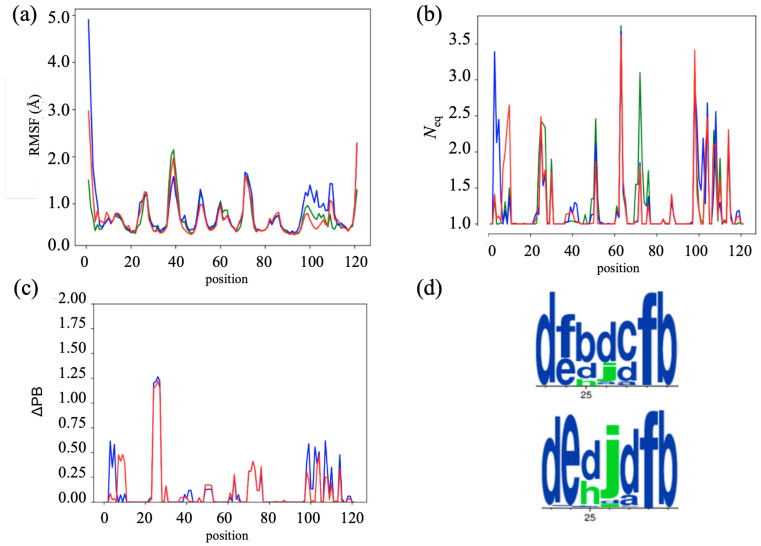
*Comparison between camelid HL6 and humanized V_H_Hs*. (**a**) RMSF and (**b**) *N*_eq_ with wild-type in green, V_H_H-h_seq_ in blue, and V_H_H-h_str_ in red. Comparison of conformational differences between the 3 systems through (**c**) ΔPB and a zoom via (**d**) WebLogo between positions 23 and 29 (PBs in green are more associated to coil conformations and in blue to extended conformations).

When comparing the camelid HL6 V_H_H with the two humanized V_H_Hs, the RMSF profiles were strikingly similar, with correlations higher than 0.9 (excluding extremities). The most significant difference in flexibility is observed in the humanized V_H_H-h_seq_ (see Figure 6a). Specifically, there was a slight decrease in flexibility in the FR2 region, with RMSF values of 1.5 Å compared to 2.0 Å in the camelid V_H_H. In addition, an increase in flexibility is observed in the CDR3 loop, with RMSF values of 1.3 Å instead of 0.8 Å in the camelid V_H_H. Minimal differences between the humanized V_H_H and the camelid V_H_H are observed in other regions.

Further analysis is then performed using protein blocks. Figure 6b illustrates the evolution of *N*_eq_ along the sequences. All three systems show a similar pattern in terms of flexibility, with a common fluctuation in the FR2, CDR2, FR3, CDR3, and FR4 regions. However, a slight decrease in flexibility is observed in the CDR1 and CDR2 loops of the two humanized V_H_Hs. In addition, the FR1 region initially displays higher flexibility in V_H_H-h_seq_, but later undergoes a transition to higher flexibility in V_H_H-h_str_. These observations suggest that humanization has a subtle effect, mainly on the first two CDR loops of the V_H_H.

As shown previously, ΔPB allows us to see precisely the difference in the occurrence of PB during dynamics (see Figure 6c). An obvious difference is located in CDR1, together with a more limited effect observed in the FR1 and CDR3 regions. For the latter two, it corresponds to the same region highlighted in the previous section, with a ΔPB value of around 0.50 (indicating that a quarter of the PB occurrences differ). In CDR1, it is specifically between positions 24 and 27, and the ΔPB values reached 1.25 for the two humanized V_H_Hs, i.e., 2/3 of the time PBs are different.

The PB differences in CDR1 are highlighted in Figure 6d with a PB WebLogo plot. ΔPB of 1.25 is a significant difference. Between positions 23 and 29, the camelid V_H_H predominantly shows the *dfbdcfb* series of PBs, while the two humanized V_H_Hs display the *dedjdfb* series. Thus, in the two humanized V_H_Hs, the classic *fbdc* loop has been replaced in a privileged way by the *edjd* loop, which is much rarer [44]. It is therefore a strict change in the dynamics of CDR1 for four residues during the simulation. In fact, it is the only one actually observed for the whole V_H_H. It should be noted that these two types of loops are quite close from a 3D point of view and should not have any real impact on the V_H_H interactions.

## 3. Materials and Methods

### 3.1. V_H_H Structure

The structure of V_H_H was downloaded from the Protein Data Bank website (https://www.rcsb.org, accessed on 29 May 2023) [34], with PDB id 1OP9 [33]. The structure was analyzed by classical approaches such as MolProbity [45] and visually with PyMOL v. 2.4.0 software (https://pymol.org/2/, accessed on 29 August 2023, Schrödinger, Inc.: New York, NY, USA) [46,47,48].

### 3.2. Humanization

Llamanade is a recent computational open-source for V_H_H humanization (https://github.com/sangzhe/Llamanade/blob/main/README.md, accessed on 29 May 2023) [27]. This tool is used in the study of the humanization impact on the V_H_Hs structure. From the V_H_H sequence or structure, the FRs and CDRs annotations are performed via Martin’s scheme [38]. After comparison of the studied V_H_H with the matrix containing the residue frequency of human VHs, some residues were selected to be mutated if they showed a threshold higher than 10%. Thus, the humanized sequence and the corresponding humanization score, which quantifies the level of humanization of V_H_Hs, are obtained for each V_H_H query. Llamanade uses the provided V_H_H structures or builds a V_H_H structural model to optimize the humanization scheme. Additionally, Llamanade can propose a structural model thanks to external software, NanoNet [30].

Mutations in V_H_Hs structure are realized by the SCWRL 4.04 [49] tool in PyMOL 2.4.0 [46,47]. SCWRL is a computer program for side-chain prediction that can be used to introduce mutations into a protein structure. This mutagenesis tool replaces one amino acid with another by converting the side chain. The SCWRL mutation is based on a library of rotamers (from PDB) that contains the frequency of rotamers according to the backbone ϕ and Ψ dihedral angles. This library also has information about the average side-chain dihedral angles based on the backbone.

### 3.3. Molecular Dynamics

Molecular dynamics (MD) simulations were conducted using GROMACS 2021.4 software [50] with the CHARMM-36 force field [51], considering three different systems: the initial PDB structure of the V_H_H and the two humanized models obtained with Llamanade. Before starting any simulation experiments, each structural variant was energy minimized for 500 steps of steepest descent and 500 steps of conjugate gradient using the Gromacs suite. V_H_H camelid structure and the two structural model forms were soaked in a rhombic dodecahedral simulation box with TIP3P water molecules. After that, charge neutralization is achieved by adding sodium and chloride ions.

The MD protocol had been standardized through our previous work [52,53]. After 1 nanosecond (nsec) of equilibration (with position restraints on the protein), each system was simulated through multiple classical independent production runs with 4 replicates of 250 nanoseconds as in [52]. The equilibration consists of one step with a NVT system and three more with a NPT system (with a gradual decrease in position constraints). During the first step in NVT and the second step in NPT, the protein is totally constrained and unable to move, while the equilibration affects the water molecules. In the third and fourth steps of NPT, constraints on proteins are slowly released. Molecular conformations were saved every 100 picoseconds for downstream analysis. This produced 1 µs of MD simulation for each system.

Trajectory analyses were conducted with the GROMACS software and in-house Python and R scripts. Root mean square deviations (RMSD) and root mean square fluctuations (RMSF) were calculated on Cα atoms only.

### 3.4. MD Analysis

The analysis of MDs is conducted using classic tools, such as RMSD and RMSF, and other more innovative ones, such as PBxplore [54], a tool developed within the team. PBxplore makes it possible to analyze protein blocks throughout the MD. RMSD (root mean square deviation) quantifies structural variations during dynamics by comparing each frame to a reference structure, here the starting frame. For each frame, an average of the differences between the reference positions and the positions of the current frame is performed in order to have an RMSD value per unit of time.

The RMSF (root mean square fluctuation) is similar to the RMSD by determining the fluctuation of each residue following the same principle as for the RMSD, i.e., a comparison with a reference. But this time, it is the average position of each residue, and thus the measure of the difference between the current position and the average position, in order to have a flexibility value per position.

The assignment of secondary structures was performed using the dictionary of secondary structure of protein (DSSP) [55,56]. DSSP provides eight states of description (α-helix, π-helix, 3_10_-helix, β-strands, β-turns, bents, β-bridge, and coil). Thus, from the trajectory file generated by GROMACS, DSSP assigns the secondary structure element for each time interval. This analysis allows us to easily see the stability or not of the protein secondary structure elements as a function of time.

Protein blocks (PBs) are a structural alphabet composed of 16 local prototypes [57,58,59]. Each specific PB is characterized by the φ, ψ dihedral angles of five consecutive residues, with each PB assignment focused on the central residue. Obtained through an unsupervised training approach and performed on a representative, non-redundant databank, PBs give a reasonable approximation of all local protein 3D structures [60]. PBs are very efficient in tasks such as protein superimpositions [61,62] and MD analyses [63,64]. They are labeled from *a* to *p*: PBs *m* and *d* can be roughly described as prototypes for α-helix and central β-strand, respectively. PBs *a* to *c* primarily represent β-strand N-caps, and PBs *e* and *f* representing β-strand C-caps; PBs *a* to *j* are specific to coils; PBs *k* and *l* to α-helix N-caps while PBs *n* to *p* to α-helix C-caps. PB assignment was carried out using our PBxplore tool (https://github.com/pierrepo/PBxplore, accessed on 29 May 2023) [54].

PB assignments are conducted for each residue in the C-domain and for every snapshot extracted from MD simulations. The equivalent number of PBs (*N*_eq_) is a statistical measurement similar to entropy that represents the average number of PBs for a residue at a given position. *N*_eq_ is calculated as follows [57]:

$$N_{\mathrm{eq}}=\exp\left(-\overset{16}{\underset{x=1}{\sum }}f_{x}\ln f_{x}\right)$$

where *f_x_* is the probability of PB *x*. A *N*_eq_ value of 1 indicates that only one type of PB is observed, while a value of 16 is equivalent to a random distribution. To underline the main differences between one system and another one for each position, the absolute difference Δ*N*_eq_ between the corresponding *N_eqs_* values was computed.

However, since the same Δ*N*_eq_ value can be obtained with different types of blocks in similar proportions, we have defined a complementary measure, ΔPB, that evaluates a change in PB profile by calculating the absolute sum of the differences for each PB between the probabilities of a PB *x* to be present in the first and the second forms (*x* goes from PB *a* to PB *p*). Δ*PB* is calculated as follows [52]:

$$∆\mathrm{PB}=\overset{16}{\underset{x=1}{\sum }}\left|\left(f_{x}^{1}-f_{x}^{2}\right)\right|$$

where *f*^1st^*_x_* and *f*^2nd^*_x_* are the percentages of occurrence of a PB *x* in the first and second systems, respectively. A value of 0 indicates perfect PB identity between the 1st and 2nd systems, while a score of 2 indicates a maximum total difference.

PBxplore also uses WebLogo to provide a dedicated PB logo output [65].

## 4. Conclusions

Humanization of V_H_Hs involves modifying a significant percentage of residues to make them suitable for potential use in humans. In this example, 10% of the residues are modified, mainly in the FRs. Molecular dynamics simulations were used to assess their potential impact on dynamic behavior. The use of classical approaches with RMSF shows that the intrinsic flexibility of V_H_Hs is preserved. Only CDR3 is affected. The use of a more local analysis with protein blocks shows that CDR3 is indeed little affected, which is essential for the interest of V_H_Hs.

Overall, the results indicate a weak effect of humanization, with CDR1 being the most pronounced. This finding is surprising considering that the mutations are distributed among different FRs. Analysis of the PB distributions shows that PB series are different for four residues, underlying a long-range effect, and due to their close proximity, the overall effect appears to be limited. Therefore, the increased conformational diversity resulting from the introduced mutations does not seem to significantly affect conformational behavior. As a result, Llamanade [27] appears to be a promising tool for humanizing V_H_Hs and making them suitable for use in humans without any major modification of the dynamic properties.

It should be noted that this is a preliminary study, and although the results are very clear and precise, they should be seen first and foremost as specific to this system. The innovative aspect of the study was that we used this approach to simulate vehicle dynamics, where we have unique experience (88 V_H_Hs tested in our previous study [32]). It raises a more complex issue: the question of interaction with the partner. Here, it seems clear that it could take place, but it would be interesting to demonstrate this using a docking approach. The future direction of this research is therefore to have more examples of V_H_Hs, but more importantly, to further explore the question of docking these humanized V_H_Hs to see if they can be used by computational approaches.

## Data Availability

Molecular dynamics trajectories are available on request.

## Supplementary Materials

The supporting information can be downloaded at: https://www.mdpi.com/article/10.3390/ijms241914586/s1.
