# Supplementary material for: Evaluation of the Potential Impact of In Silico Humanization on VHH Dynamics
Source: Int J Mol Sci. 2023 Sep 26;24(19):14586. doi: 10.3390/ijms241914586 (PMC10572902; doi:10.3390/ijms241914586)
Supplement: Supplementary file 1 [file ijms-24-14586-s001.zip › ijms-2595904-supplementary.pdf]

Title: Evaluation of the potential impact of in silico humanization on V_HH dynamics.

Authors: Carla Martins, Julien Diharce, Aravindan Arun Nadaradjane* &
Alexandre G. de Brevern*

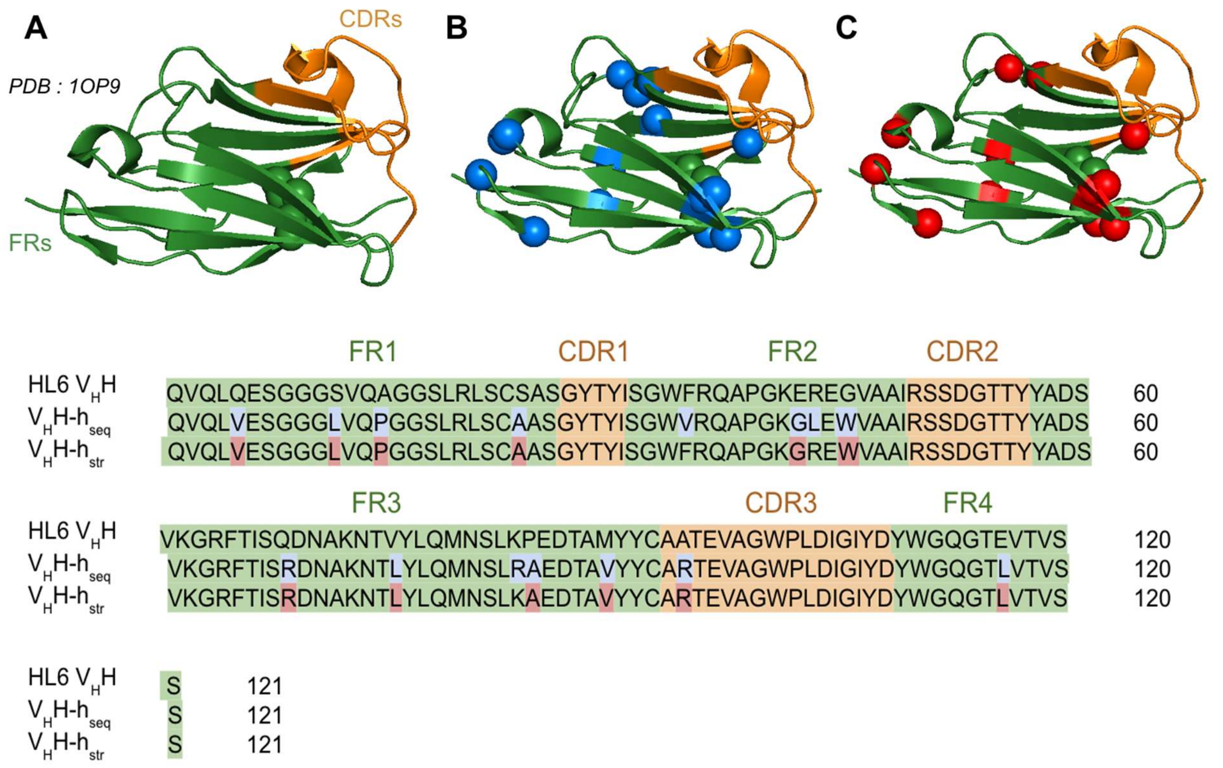

Figure S1. *HL6 VHH*. A. X-ray structure of HL6 VHH [1]. B. VHH structural model from the sole information of the sequence, modelled by NanoNet [2] (VHH-hseq). C. VHH structural model from the X-ray structure (VHH-hstr), and, (up) the 3D information, mutations are noted in blue in B and in red in C, (down) multiple alignment with the corresponding sequences (mutated positions are with their respective colours).

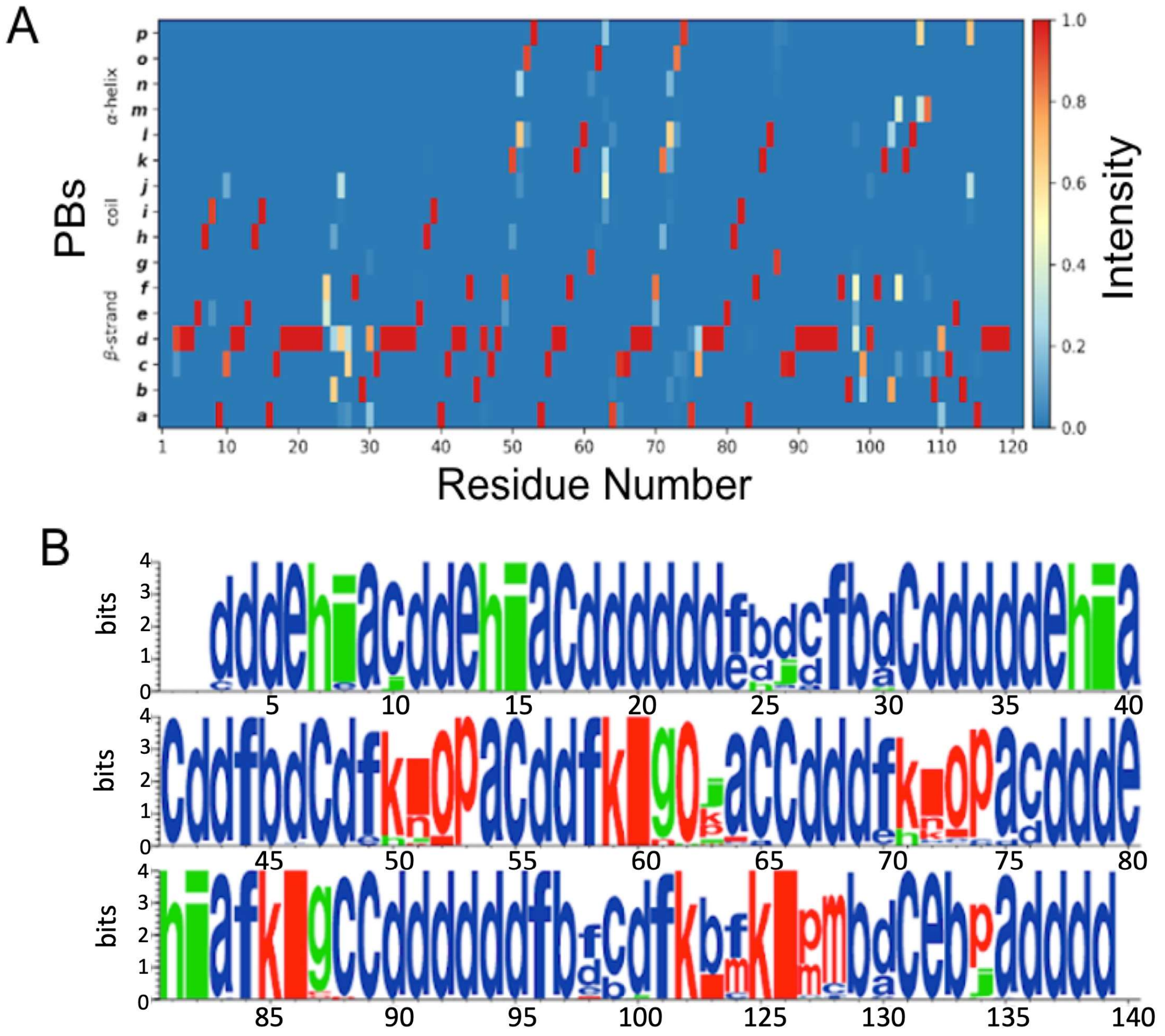

Figure S2. *Analysis of the dynamics of HL6 VHH in the light of Protein Blocks.* (a) Protein Block occurrence map [3,4], and (b) corresponding WebLogo description [5]. The height of the letter indicates the PB frequency observed at this position during MD simulations.

References

1. Dumoulin, M.; Last, A.M.; Desmyter, A.; Decanniere, K.; Canet, D.; Larsson, G.; Spencer, A.; Archer, D.B.; Sasse, J.; Muyldermans, S., *et al.* A camelid antibody fragment inhibits the formation of amyloid fibrils by human lysozyme. *Nature* **2003**, *424*, 783-788.
2. Cohen, T.; Halfon, M.; Schneidman-Duhovny, D. Nanonet: Rapid and accurate end-to-end nanobody modeling by deep learning. *Front. Immunol* **2022**, *13*, 958584.
3. de Brevern, A.G.; Etchebest, C.; Hazout, S. Bayesian probabilistic approach for predicting backbone structures in terms of protein blocks. *Proteins* **2000**, *41*, 271-287.
4. Barnoud, J.; Santuz, H.; Craveur, P.; Joseph, A.P.; Jallu, V.; de Brevern, A.G.; Poulain, P. Pbxplore: A tool to analyze local protein structure and deformability with protein blocks. *PeerJ* **2017**, *5*, e4013.
5. Crooks, G.E.; Hon, G.; Chandonia, J.M.; Brenner, S.E. Weblogo: A sequence logo generator. *Genome Res.* **2004**, *14*, 1188-1190.
